# Supplementary material for: Substrate stiffness modulates phenotype-dependent fibroblast contractility and migration independent of TGF-β stimulation
Source: Mechanobiol Med. 2025 Sep 24;3(4):100158. doi: 10.1016/j.mbm.2025.100158 (PMC12529497; doi:10.1016/j.mbm.2025.100158)
Supplement: Multimedia component 1 [file mmc1.pdf]

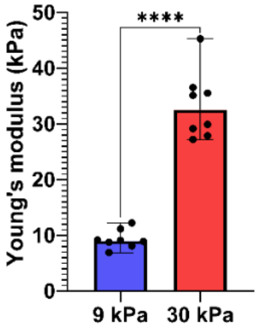

Figure S1. Young's modulus of pAA hydrogels. ***** p<0.0001(Unpaired t test)

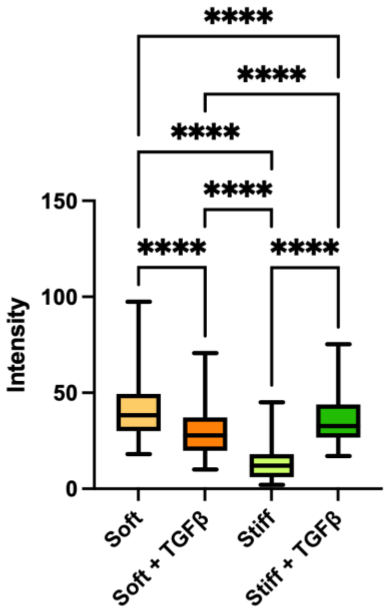

Figure S2. Mean image fluorescence intensity of cytoplasmic αSMA in fibroblasts cultured on hydrogels with different stiffnesses, with and without TGF-β. Data for each experimental group were obtained from analysis of >5 images from 3 independent samples and shown as median and IQR with min and max values. ****p < 0.0001.

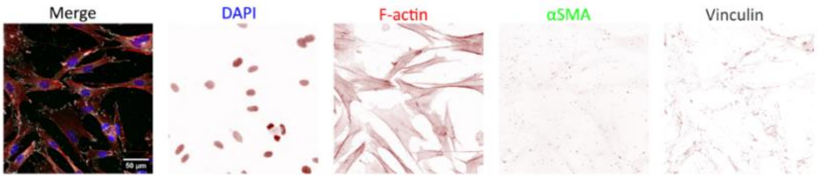

Figure S3. Fibroblasts cultured 96 hours on glass substrate in presence of AIIB2 were stained for DAPI (blue), αSMA (green), f-actin (red) and vinculin (grey). Scale bar = 50 μm

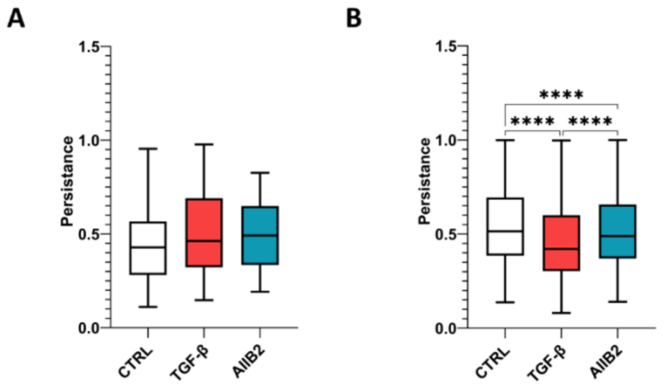

Figure S4. Fibroblast persistence shows differences in different phenotypical states. A-B) Persistence of fibroblasts cultured on glass for (A) 48 hours and (B) 96 hours. **** p<0.0001 (One-way ANOVA)

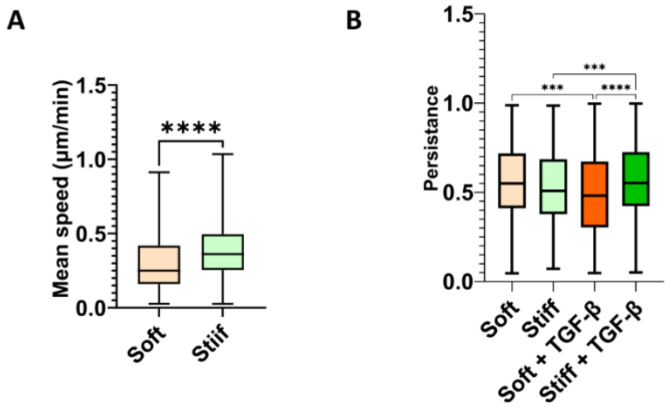

Figure S5. A) Mean speed of fibroblasts cultured on soft and stiff hydrogels for 48 hours. **B)** Persistence of fibroblast cultured on pAA hydrogels for 96 hours. ***p<0.005 , ****p<0.0001 (Unpaired t-test and One-way ANOVA)
